# Supplementary material for: The Impact of Regular Physical Exercise on Psychopathology, Cognition, and Quality of Life in Patients Diagnosed with Schizophrenia: A Scoping Review
Source: Behav Sci (Basel). 2023 Nov 21;13(12):959. doi: 10.3390/bs13120959 (PMC10740550; doi:10.3390/bs13120959)
Supplement: Supplementary file 1 [file behavsci-13-00959-s001.zip › behavsci-2660183-File S1.pdf]

Supplementary File S1: Electronic search strategy

Electronic databases	Search equation	Number of results
Web of Science (WOS)<br><br>URL: https://www.webofscience.com/wos/woscc/summary/0be7fa17-7dd3-4125-b29a-a38b879bdcb3-6f07d0f9/relevance/1<br><br>Dates: from 1982 to April 2022	("power exercise*" OR "power train*" OR "strength exercise*" OR "strength train*" OR "resistance train*" OR "resistance exercise*" OR "exercise program" OR "endurance train*" OR "endurance exercise" OR "physical exercise*" OR "plyometric*" OR "aerobic exercise*" OR "aerobic train*" OR "anaerobic exercise*" OR "anaerobic train*") AND ("schizo*") (Topic) and English or Spanish (Languages)	393
Scopus<br><br>URL:<br>https://www-scopus-com.accedys.udc.es/results/results.uri?sort=plf-f&src=s&st1=%22power+exercise*%22+OR+%22power+train*%22+OR+%22strength+exercise*%22+OR+%22strength+train*%22+OR+%22resistance+train*%22+OR+%22resistance+exercise*%22+OR+%22exercise+program%22+OR+%22endurance+train*%22+OR+%22endurance+exercise*+%22+OR+%22physical+exercise*%22+OR+%22plyometric*%22+OR+%22aerobic+exercise*%22+OR+%22aerobic+train*%22+OR+%22anaerobic+exercise*%22+OR+%22anaerobic+train*%22&st2=%22schizo*%22&nlo=&nlr=&nls=&sid=10eb65111054c1a8e99e173938fd4f7e&sot=b&sdt=cl&cluster=scolang%2c%22English%22%2ct%2c%22Spanish%22%2ct&sl=376&s=%28TITLE-ABS-KEY%28%22power+exercise*%22+OR+%22power+train*%22+OR+%22strength+exercise*%22+OR+%22strength+train*%22+OR+%22resistance+train*%22+OR+%22resistance+exercise*%22+OR+%22exercise+program%22+OR+%22endurance+train*%22+OR+%22endurance+exercise*+%22+OR+%22physical+exercise*%22+OR+%22plyometric*%22+OR+%22aerobic+exercise*%22+OR+%22aerobic+train*%22+OR+%22anaerobic+exercise*%22+OR+%22anaerobic+train*%22%29+AND+TITLE-ABS-KEY%28%22schizo*%22%29%29&origin=resultslist&zone=leftSideBar&editSaveSearch=&txGid=360433d0ef871f69b19b804f0b4fcad9<br><br>Dates: from 1968 to April 2022	(TITLE-ABS-KEY ("power exercise*" OR "power train*" OR "strength exercise*" OR "strength train*" OR "resistance train*" OR "resistance exercise*" OR "exercise program" OR "endurance train*" OR "endurance exercise* " OR "physical exercise*" OR "plyometric*" OR "aerobic exercise*" OR "aerobic train*" OR "anaerobic exercise*" OR "anaerobic train*") AND TITLE-ABS-KEY ("schizo*")) AND (LIMIT-TO (LANGUAGE , "English") OR LIMIT-TO (LANGUAGE , "Spanish"))	360

PubMed<br><br>URL:<br>https://pubmed.ncbi.nlm.nih.gov/?term=%28%22power+exercise*%22+OR+%22power+train*%22+OR+%22strength+exercise*%22+OR+%22strength+train*%22+OR+%22resistance+train*%22+OR+%22resistance+exercise*%22+OR+%22exercise+program%22+OR+%22endurance+train*%22+OR+%22endurance+exercise*%22+OR+%22physical+exercise*%22+OR+%22plyometric*%22+OR+%22aerobic+exercise*%22+OR+%22aerobic+train*%22+OR+%22anaerobic+exercise*%22+OR+%22anaerobic+train*%22%29+AND+%28%22schizo*%22%29&filter=lang.english&filter=lang.portuguese&filter=lang.spanish<br><br>Dates: from 1968 to April 2022	("power exercise*" OR "power train*" OR "strength exercise*" OR "strength train*" OR "resistance train*" OR "resistance exercise*" OR "exercise program" OR "endurance train*" OR "endurance exercise*" OR "physical exercise*" OR "plyometric*" OR "aerobic exercise*" OR "aerobic train*" OR "anaerobic exercise*" OR "anaerobic train*") AND ("schizo*") AND (english[Filter] OR portuguese[Filter] OR spanish[Filter])	222
SPORTDiscus<br><br>URL:<br>https://search.ebscohost.com/login.aspx?direct=true&AuthType=ip,uid&db=s3h&bquery=(+%26quot%3bpower+exercise*%26quot%3b+OR+%26quot%3bpower+train*%26quot%3b+OR+%26quot%3bstrength+exercise*%26quot%3b+OR+%26quot%3bstrength+train*%26quot%3b+OR+%26quot%3bresistance+train*%26quot%3b+OR+%26quot%3bresistance+exercise*%26quot%3b+OR+%26quot%3bexercise+program%26quot%3b++OR+%26quot%3bendurance+train*%26quot%3b+OR+%26quot%3bendurance+exercise%26quot%3b+OR+%26quot%3bphysical+exercise*%26quot%3b+OR+%26quot%3bplyometric*%26quot%3b+OR+%26quot%3baerobic+exercise*%26quot%3b+OR+%26quot%3baerobic+train*%26quot%3b+OR+%26quot%3banerobic+exercise*%26quot%3b+OR+%26quot%3banaerobic+train*%26quot%3b+)+AND+%26quot%3bschizo*%26quot%3b&lang=es&type=1&searchMode=Standard&site=ehost-live<br><br>Dates: from 1990 to April 2022	("power exercise*" OR "power train*" OR "strength exercise*" OR "strength train*" OR "resistance train*" OR "resistance exercise*" OR "exercise program" OR "endurance train*" OR "endurance exercise* " OR "physical exercise*" OR "plyometric*" OR "aerobic exercise*" OR "aerobic train*" OR "anaerobic exercise*" OR "anaerobic train*”) AND "schizo*"<br><br>With English and Portuguese language filters	21
