# Supplementary material for: The Impact of Regular Physical Exercise on Psychopathology, Cognition, and Quality of Life in Patients Diagnosed with Schizophrenia: A Scoping Review
Source: Behav Sci (Basel). 2023 Nov 21;13(12):959. doi: 10.3390/bs13120959 (PMC10740550; doi:10.3390/bs13120959)
Supplement: Supplementary file 1 [file behavsci-13-00959-s001.zip › behavsci-2660183-File S2.pdf]

Supplementary File S2: Excluded studies and reasons for exclusion

Did not report enough parameters for the replicability of the exercise protocol.

1. Bhatia T, Mazumdar S, Wood J, et al. A randomised controlled trial of adjunctive yoga and adjunctive physical exercise training for cognitive dysfunction in schizophrenia. Acta Neuropsychiatr. 2017;29(2):102-114. https://doi.org/10.1017/neu.2016.42. [77]
2. Nasution NM, Effendy E, Amin MM, Siregar IR. Effect of aerobic exercise in positive and negative symptoms in schizophrenia. Open Access Maced J Med Sci. 2021;9(T3):178-181. https://doi.org/10.3889/oamjms.2021.6324. [78]
3. Wang PW, Lin HC, Su CY, et al. Effect of aerobic exercise on improving symptoms of individuals with schizophrenia: A single blinded randomized control study. Front Psychiatry. 2018;9(MAY). https://doi.org/10.3389/fpsyt.2018.00167. [68]
4. Malchow B, Keeser D, Keller K, et al. Effects of endurance training on brain structures in chronic schizophrenia patients and healthy controls. Schizophr Res. 2016;173(3):182-191. https://doi.org/10.1016/j.schres.2015.01.005. [79]
5. Chen MD, Kuo YH, Chang YC, Hsu ST, Kuo CC, Chang JJ. Influences of Aerobic Dance on Cognitive Performance in Adults with Schizophrenia. Occup Ther Int. 2016;23(4):346-356. https://doi.org/10.1002/oti.1436. [80]
6. Sisman FN, Büber B, Taş F, Turan H. Randomized Controlled Trial for the Effects of an Exercise Program for Functional Remission and Weight Control in Schizophrenia: A Community Mental Health Study. Issues Ment Health Nurs. Published online January 28, 2022:1-10. https://doi.org/10.1080/01612840.2021.2024630. [81]
7. Maurus I, Mantel C, Keller-Varady K, et al. Resistance training in patients with schizophrenia: Concept and proof of principle trial. Journal of Psychiatric Research. 2020;120:72-82. https://doi.org/10.1016/j.jpsychires.2019.09.015. [62]
8. Acil AA, Dogan S, Dogan O. The effects of physical exercises to mental state and quality of life in patients with schizophrenia. J Psychiatr Ment Health Nurs. 2008;15(10):808-815. https://doi.org/10.1111/j.1365-2850.2008.01317.x. [24]
9. Le TP, Ventura J, Ruiz-Yu B, McEwen SC, Subotnik KL, Nuechterlein KH. Treatment engagement in first-episode schizophrenia: Associations between intrinsic motivation and attendance during cognitive training and an aerobic exercise program. Schizophr Res. 2023;251:59-65. https://doi.org/10.1016/j.schres.2022.12.018. [82]
10. Nuechterlein KH, McEwen SC, Ventura J, et al. Aerobic exercise enhances cognitive training effects in first-episode schizophrenia: randomized clinical trial demonstrates cognitive and functional gains. Psychol Med. 2023;53(10):4751-4761. https://doi.org/10.1017/S0033291722001696. [83]

Did not include a control group with no exposure to another intervention.

11. Ventura J, McEwen S, Subotnik KL, et al. Changes in inflammation are related to depression and amount of aerobic exercise in first episode schizophrenia. Early Intervent Psychiatry. 2021;15(1):213-216. https://doi.org/10.1111/eip.12946. [84]
12. Nuechterlein KH, Ventura J, McEwen SC, Gretchen-Doorly D, Vinogradov S, Subotnik KL. Enhancing Cognitive Training Through Aerobic Exercise After a First Schizophrenia Episode: Theoretical Conception and Pilot Study. Schizophr Bull. 2016;42 Suppl 1(Suppl 1):S44-52. https://doi.org/10.1093/schbul/sbw007. [85]
13. Bang-Kittilsen G, Egeland J, Holmen TL, et al. High-intensity interval training and active video gaming improve neurocognition in schizophrenia: a randomized controlled trial. Eur Arch Psychiatry Clin Neurosci. 2021;271(2):339-353. https://doi.org/10.1007/s00406-020-01200-4. [86]
14. Cristiano VB, Szortyka MF, Belmonte-de-Abreu P. A controlled open clinical trial of the positive effect of a physical intervention on quality of life in schizophrenia. Front Psychiatry. 2023;14:1066541. Published 2023 Feb 24. https://doi.org/10.3389/fpsyt.2023.1066541. [87]

Data from a study protocol.

15. Arietaleanizbeaskoa MS, Sancho A, Olazabal I, et al. Effectiveness of physical exercise for people with chronic diseases: the EFIKRONIK study protocol for a hybrid, clinical and implementation randomized trial. BMC Fam Pract. 2020;21(1). https://doi.org/10.1186/s12875-020-01298-4. [88]

Did not apply an intervention with physical exercise.

16. Nyboe L, Moeller MK, Vestergaard CH, Lund H, Videbech P. Physical activity and anomalous bodily experiences in patients with first-episode schizophrenia. Nord J Psychiatry. 2016;70(7):514-520. https://doi.org/10.1080/08039488.2016.1176250. [89]

Data from population that did not meet the established inclusion criteria.

(A) *Did not include outpatients.*

17. Shimada T, Ito S, Makabe A, Yamanushi A, Takenaka A, Kobayashi M. Aerobic exercise and cognitive functioning in schizophrenia: A pilot randomized controlled trial. Psychiatry Res. 2019;282. https://doi.org/10.1016/j.psychres.2019.112638. [90]
18. Shimada T, Ito S, Makabe A, et al. Aerobic exercise and cognitive functioning in schizophrenia: Results of a 1-year follow-up from a randomized controlled trial. Psychiatry Res. 2020;286. https://doi.org/10.1016/j.psychres.2020.112854. [91]
19. Khonsari NM, Badrfam R, Mohammdi MR, et al. Effect of Aerobic Exercise as Adjunct Therapy on the Improvement of Negative Symptoms and Cognitive Impairment in Patients With Schizophrenia: A Randomized, Case-Control Clinical Trial. J Psychosoc Nurs Ment Health Serv. Published online October 25, 2021:1-7. https://doi.org/10.3928/02793695-20211014-03. [92]
20. Şenormancı G, Korkmaz N, Şenormancı Ö, Uğur S, Topsaç M, Gültekin O. Effects of Exercise on Resilience, Insight and Functionality in Patients with Chronic Schizophrenia in a Psychiatric Nursing Home Setting: A Randomized Controlled Trial. Issues Ment Health Nurs. 2021;42(7):690-698. https://doi.org/10.1080/01612840.2020.1847221. [93]
21. Heggelund J, Nilsberg GE, Hoff J, Morken G, Helgerud J. Effects of high aerobic intensity training in patients with schizophrenia - A controlled trial. Nord J Psychiatry. 2011;65(4):269-275. https://doi.org/10.3109/08039488.2011.560278. [94]
22. Maggouritsa G, Kokaridas D, Theodorakis I, et al. The effect of a physical activity programme on improving mood profile of patients with schizophrenia. Int J Sport Exerc Psychol. 2014;12(3):273-284. https://doi.org/10.1080/1612197X.2014.898968. [95]
23. Areshtanab HN, Ebrahimi H, Abdi M, Mohammadian R, Asl AM, Piri S. The effect of aerobic exercise on the quality of life of male patients who suffer from chronic schizophrenia: Double-blind, randomized control trial. Iran J Psychiatr Behav Sci. 2021;14(4). https://doi.org/10.5812/ijpbs.67974. [96]
24. Areshtanab H, Ebrahimi H, Farnam A, Mohammadpoorasl A, Jamali B, Piri S. The effect of regular aerobic exercise on both positive and negative symptoms of male patients with chronic Schizophrenia: A double blinded study. International Journal of Medical Research & Health Sciences. 2016;5(11):529-535. [97]
25. Ho RTH, Wan AHY, Au-Yeung FSW, et al. The psychophysiological effects of Tai-chi and exercise in residential Schizophrenic patients: A 3-arm randomized controlled trial. BMC Complement Altern Med. 2014;14(1). https://doi.org/10.1186/1472-6882-14-364. [98]
26. Heggelund J, Morken G, Helgerud J, Nilsberg GE, Hoff J. Therapeutic effects of maximal strength training on walking efficiency in patients with schizophrenia - A pilot study. BMC Res Notes. 2012;5. https://doi.org/10.1186/1756-0500-5-344. [61]

(B) *Data from different pathologies without analysis subgroup of schizophrenia.*

27. Mullor D, Gallego J, Cangas A, Aguilar-Parra J, Trigueros R, Lopez-Pardo A. Psychological and social impact of an inclusive sports program among students and people with severe mental disorder. Revista de Psicologia del Deporte. 2020;29:8-15. [99]
28. Hasson-Ohayon I, Kravetz S, Roe D, Rozencwaig S, Weiser M. Qualitative assessment of verbal and non-verbal psychosocial interventions for people with severe mental illness. J Ment Health. 2006;15(3):343-353. https://doi.org/10.1080/09638230600700847. [100]
